# A needle-form 3-omega sensor for thermal conductivity measurements of soft materials and biological tissues

**DOI:** 10.1038/s41598-025-26808-1

**Published:** 2025-11-28

**Authors:** Spencer P. Alliston, Chris Dames

**Affiliations:** Department of Mechanical Engineering, University of California, Berkeley, CA 94720 USA

**Keywords:** Engineering, Materials science, Physics

## Abstract

Soft materials, liquids, and biological tissues are of increasing interest as thermal materials for applications in energy storage, electrical/electrochemical systems, and cryopreservation, but thermal characterization of these materials can be challenging experimentally. Here, we extend the robust 3-omega method, which is traditionally based on a planar form factor with external sample contact, to a microfabricated needle-form sensor that can be plunged directly into a sample. We further demonstrate the reusability of this sensor, the ability to easily make thermal contact by plunging the sensor into the center of a sample, and the ability to sample systems undergoing phase transformations. We do so via application to solid ice as well as 4 representative materials at room temperature: water, glycerol, paraffin, and chicken liver, thereby demonstrating the sensor’s utility for liquids, soft solid materials, and phase change materials. Data analysis is conducted by fitting to a three dimensional numerical model of the sensor and sample. These experiments show very good agreement of within 3% of literature values for thermal conductivity for the explored materials, which range in thermal conductivity from approximately 0.3 W/mK to 2.3 W/mK.

## Introduction

The thermal transport properties of soft materials, liquids, and biological tissues are important to a wide array of fields, including cryopreservation^1^, thermal energy storage using phase change materials^2^, interfacial material design^3^, and more. Broad understanding of these samples, especially under experimental or operational conditions, is limited by the lack of readily distributed characterization methods. This is particularly deleterious for the case of biological tissues, which can be highly heterogeneous in nature^4^. Many measurements are required to better understand and model the thermal behavior of such biological systems, which includes critical inputs for modeling the physics of cryosurgery^5,6^ and cryopreservation of diverse tissues and organisms^1^.

The transient hot wire method has been used extensively for thermal characterization of liquids^7–9^. However, this method requires extremely thin (or long) wire^8^ which may be too fragile for measuring many types of soft materials. Liang et al. have adapted this method into a microfabricated needle-form sensor^10^ for measurement of semi-rigid tissues; this configuration is also well-suited for the applications we identify below in this work, but has not been adapted into a frequency-domain technique which offers several advantages^11^. Included among these are a lower necessary heat input, which improves the temperature resolution of measurements and allows for measurements of temperature-sensitive samples, and increased control over the thermal penetration depth, which allows for measurement of smaller samples and the suppression of convective heat transfer and is further discussed in the Supplementary Information.

To this end, we here present a novel needle-form sensor allowing for the implementation of a modified 3-omega method for measuring the thermal conductivity of soft samples. We previously gave a preliminary demonstration of this sensor for a single sample of cryopreserved tissue in the solid state^12^; and now give a detailed study of the sensor’s design, the data analysis, and finally demonstrate its broader application to a variety of soft and liquid materials.

The 3-omega method is a well-established electrothermal technique based on sinusoidal joule heating and simultaneous temperature measurement from a single metal line. This technique has been used extensively for bulk thermal conductivity measurements of rigid solid materials^13,14^ and thin films^15,16^. More recent works, including the ’bi-directional’ 3-omega method, have extended these capabilities into soft materials^17–19^ and liquids^20–22^. Other methods to implement the 3-omega method on liquids and soft materials include using thin, compliant substrates^23,24^ or depositing the sensor directly on human skin^25^. However, each of these configurations is limited to measurement of properties at the surface of the system, and each may not be well-suited to some experimental settings. A needle-form sensor allows for the measurement of the center of samples of interest (such as the interior of biological samples) and avoids the need for a custom-fabricated stage, allowing for simpler experimental design and more varied boundary conditions of the sample (such as a sample suspended in oil).

This reusable needle-form 3-omega sensor, which uses a microfabricated needle with an integrated electrical line in the form of a neural probe, is demonstrated and evaluated on a range of relevant samples. This sensor is designed for application to soft materials, biological tissues, and liquids, all of which are represented in proof-of-concept measurements in this study. In addition to the experimental simplicity of a needle-form sensor, it has the benefits of being electrically insulated from the sample, which is important for systems where electron or ion transport occurs, and mechanically robust, which allows for reasonable stresses to be withstood in the set up and operation of experiments.

This mechanical stability also enables the simple application to liquids and soft materials, as the needle-form sensor can be plunged into the sample and affixed; this additionally allows for measurements of the interior portions of heterogeneous samples. Furthermore, it can withstand stresses such as those occurring during liquid-solid phase transformations; this is demonstrated below by measurements of crystalline ice and solid paraffin wax, which were solidified after inserting the needle into the sample in the liquid state. This indicates the ability of such a sensor to serve as an *in situ* method for thermal conductivity measurements in systems which undergo phase transformations or other mild chemical changes, such as during the cycling of thermal storage media^26^ or the degradation of electrolytes in batteries^27^.

## Results

To evaluate the performance of these sensors, we procured several sensors and developed a computational model which could process the raw experimental data to determine the sample thermal conductivity. We then took measurements of five representative samples including liquids, soft materials, biological tissues, and phase change materials: liquid water, ice Ih, glycerol, chicken liver, and paraffin wax.

From these experiments we find that a needle-form 3-omega sensor is suitable for thermal conductivity measurements of these samples using the following design and experimental procedure. For repeated measurements on a single sample with a single sensor, the sensor had a typical variance of 0.4% in thermal conductivity, and came within 3% of accepted literature values for all samples.

**Fig. 1:**
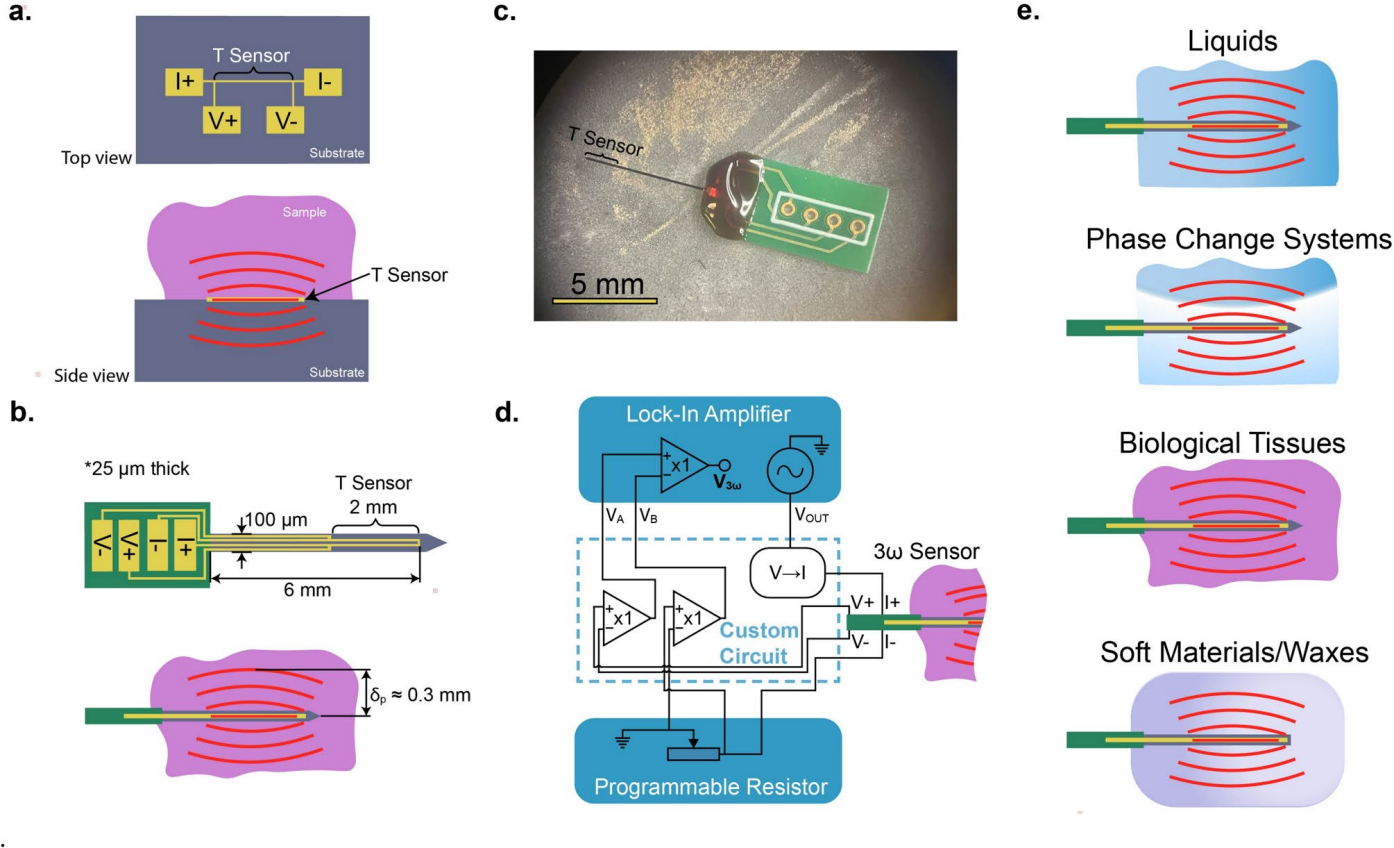
(**a**) Schematic of a traditional microfabricated 3-omega sensor of planar form factor, in top- and side-view. This requires measurement via external-contact with soft materials/biological tissues, meaning only a fraction of the sensor’s heat flows usefully into the sample, whereas much of the heat is lost through conduction into the substrate. (**b**) Schematic of the needle-form sensor as fabricated and implemented in this work, which enables the plunge-immersion internal contact for soft materials/biological tissues, minimizing heat lost to the sensor itself. The design uses a standard four point probe electrical geometry with a single bend near the tip of the needle. This immersion geometry is also more sensitive to the tissue properties by allowing heat to flow into the sample from all sides of the 3-omega sensor. Red lines indicate the decaying temperature waves inside the sample in response to periodic heating by the needle, with a typical thermal penetration depth $\delta _p$ annotated. (**c**) Microscope image of a needle-form 3-omega sensor as received before making electrical connections. (**d**) Simplified circuit diagram and experimental configuration for measurements in this work, comprising a custom 3$\omega$ circuit, a lock-in amplifier, and programmable resistor. (**e**) Application space for needle-form 3-omega sensor. Panels (**a-c**) are adapted and expanded from Alliston & Dames^12^.

### Needle-form 3-omega sensor

Figure 1a shows a simple schematic of a traditional 3-omega sensor fabricated on a rigid substrate^13,14^, as well as a ’bi-directional’ measurement configuration^1,20^, which is typical for measurement of soft or liquid samples. This electrical four point probe design allows for decoupling of the input current, which heats the sample periodically, and measurement of the voltage, which corresponds to the temperature rise of the line via the temperature coefficient of resistivity. As this measurement is based on the temperature rise of the line, Fig. 1 uses the label ’T Sensor’ to indicate the 2 mm long section of the fabricated line along which the temperature is measured.

Figure 1b shows the implemented electrical design of the needle-form sensor used in this work. The sensor has a similar four probe design as the traditional 3-omega method (Fig 1a), which allows for extended heating down the shank of the needle while limiting *T* measurement to the probed section at the tip between $V+$ and $V-$. Fabrication of the needle-form sensor was performed by NeuroNexus, Inc., using standard fabrication techniques for neural probes, with customization here to enable the electrothermal measurements that are the foundation of the 3-omega method. The shank of the needle is made of silicon, and the electrical line is predominantly gold. A dielectric layer insulates the line from the sample. Fig 1c shows a microscope image of the sensor, Fig 1d shows the experimental configuration used to take measurements, and Fig 1e illustrates possible applications of this needle-form sensor, each of which is demonstrated in this work.

The needle is rectangular in cross section (25 ${\upmu }\hbox {m}$ x 100 ${\upmu }\hbox {m}$), and is 6 mm long. The sensing portion of the needle is 2 mm in length. Assuming the sensor is to be fully immersed in the sample, and accounting for a thermal penetration depth $\delta _p$ of $\sim$ 0.1–0.3 mm at typical measurement frequencies, this design is suitable for measuring the bulk conductivity of samples that are at least 7 mm in length (parallel to the needle axis) and 0.5 mm in thickness and width (directions normal to the needle axis). More details on sensor geometry can be found accompanying Supplemental Figure 2.

The sensor is strong enough that it can be inserted into soft materials, as here demonstrated for chicken liver, without any other mechanical supports. Similarly, this robustness allows it to withstand liquid-solid phase transformations, which we use in preparing the samples for ice and paraffin measurements.

In contrast to typical 3-omega techniques, which involve custom fabrication of the metal heater line on a substrate of interest, and bi-directional 3-omega, which requires application and maintenance of a dielectric layer when working with electrically-active samples^20^ and similarly is substrate supported and external to the sample, this needle-form sensor is fully reusable and electrically insulated from the sample interior. Between measurements, sensors are washed with isopropyl alcohol and deionized water. Measurements in this study were taken on 3 sensors. Sensor 1 was used to measure water, glycerol, and paraffin. Sensor 2 was used to measure water and chicken liver. Sensor 3 was used to measure ice. This is particularly useful as 3-omega sensors require characterization before being used to determine the temperature coefficient of resistivity, $\alpha$. For the sensors used in this study, the measured $\frac{d R}{d T}$ values were within 3% of one another, as can be seen in Supplemental Figure 1. Because the calculated value of the temperature rise scales inversely with this parameter, it may be possible to use an average value instead of individually characterizing without introducing excessive errors.

**Fig. 2:**
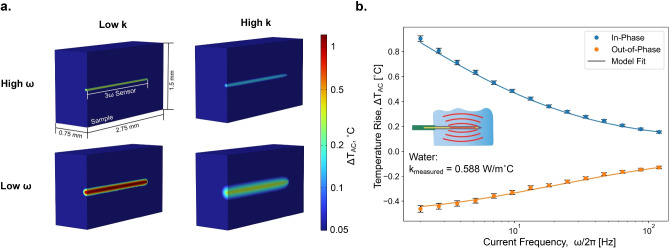
Data analysis for 3-omega method adapted for use with needle-form sensor. a) Representative in-phase temperature maps of temperature-sensing portion of sensor and surrounding sample from the 3-D comsol model used in analysis, shown for high and low frequency, $\omega$, and sample thermal conductivity, k. Thermal conductivities are representative of the lowest and highest conductivities measured in this study (0.28 $\hbox {W/m}^{\circ }\hbox {C}$ and 2.27 $\hbox {W/m}^{\circ }\hbox {C}$); For ease of visualization, frequencies are representative of the lowest and center frequencies used in measurement ($\omega /2\pi$=2.001 Hz and 18.95 Hz). The central linear shape in each image is the 2-mm sensing portion of the needle-form sensor, and the surrounding area is the measured sample. b) Data from a typical measurement using the needle-form 3-omega sensor (water, $21^{\circ }\hbox {C}$, expected k = 0.601 $\hbox {W/m}^{\circ }\hbox {C}$^28^). Points represent the measured voltage converted to the average temperature rise of the metal line within the needle, with error bars representing the propagated error of the measurement. Solid lines represent the best fit provided by the comsol model. Similar plots for each sample and details on propagated error are included in the Supplementary Information. The model fit is in excellent agreement with the experiment across all frequencies, and returns a best-fit value of k that is within 3% of the literature value.

### Data analysis

Data are analyzed by way of a fitting algorithm connected to a 3-D numerical comsol model of the periodic heat transfer in a needle-form sensor + sample system. Typical temperature map results of the comsol model are shown in in Fig. 2a. For lower frequency simulations, the temperature rise of the sensor is higher and heat penetrates further into the sample. Higher sample thermal conductivities cause a similar increase in heat spreading but result in a lower temperature rise.

The heat spreading is characterized by the thermal penetration depth $\delta _p$ within the sample,

1 $$\begin{aligned} \delta _p = \sqrt{\frac{k}{2\omega C}}, \end{aligned}$$

where *k* and *C* are the sample’s thermal conductivity and volumetric heat capacity, respectively, and $2\omega$ [rad/s] is the angular frequency of the heat input. As an electrothermal method based on joule heating, this $2\omega$ is double the frequency of the AC electrical input $\omega$.

A representative set of experimental data are shown by the points in Fig. 2.b. Each point represents the 30 second average of the voltage of the third harmonic, which corresponds to the temperature rise of the temperature sensor (“T Sensor” in Fig. 1) through^15^,

2 $$\begin{aligned} \Delta T_{AC} = \frac{2V_{3\omega }}{\alpha IR}, \end{aligned}$$

where $\Delta T_{AC}$ is the amplitude of the oscillating temperature rise of the sensor associated with the AC power input, $V_{3\omega }$ is the amplitude of the third harmonic of the measured voltage across the sensor, $\alpha =\frac{1}{R}\frac{dR}{dT}$ is the temperature coefficient of resistance of the gold line in the sensor, *I* is the amplitude of the AC input current, and *R* is the electrical resistance of the temperature sensor. In evaluating this $\Delta T_{AC}$ expression, $V_{3\omega }$, *I*, and *R* are measured directly during every experiment, with $\frac{dR}{dT}$ determined separately for each sensor through a separate calibration (see Supplemental Figure 1).

To extract a sample’s *k* from raw frequency-sweep data as in Fig. 2b requires a thermal model. For sufficiently simple geometries, like an infinitely long line heater on a semi-infinite substrate, the solution is known analytically^29,30^. The needle-form geometry used here is more complex and requires numerical analysis, which we implement using the comsol finite element package. The model in comsol has the same geometry and thermal behavior as the needle-form 3-omega sensor, and the sample of interest has variable thermal conductivity and volumetric heat capacity. More details on the comsol model can be found in Supplemental Table 1 and Supplemental Figure 2.

For every set of frequency-sweep measurements (as in Fig. 2b) the data are fed to an non-linear optimization algorithm in matlab, which then fits the comsol model to the data by varying the thermal conductivity and heat capacity of the surrounding sample. The outputs of the code are the best-fit values of (*k*, *C*) as well as the best-fit curves $\Delta T_{AC}(\omega )$ (e.g. the two lines in Fig.2b) and the corresponding minimized residual error (typically $\sim 0.01^{\circ }\hbox {C}$). The model has one other parameter, a thermal resistor $R_\text {shank}$ representing the parasitic heat loss along the shank of the needle to its base. The value $R_\text {shank}=3,200 ^{\circ }\hbox {C}/\hbox {W}$ was determined empirically by using glycerol as a standard reference sample; glycerol was chosen for this calibration because reliable literature data is available^31^ and also because it has the lowest *k* among these samples, which makes it most sensitive to any such parasitic losses. For future applications aimed at measuring samples of even lower thermal conductivity this may have to be further characterized; a discussion on this can be found accompanying Supplemental Table 1. After thus fixing $R_\text {shank}$ it was held constant for all other samples.

While this fitting scheme in principle is able to extract both thermal conductivity and heat capacity, we find in practice that the errors for heat capacity are large when compared to accepted literature values. The present method is less sensitive to heat capacity as demonstrated by a representative sensitivity study in Supplemental Figure 3, and AC heat capacity measurements in general are known to be susceptible to noise associated with thermal non-idealities, such as small changes in temperature or contact resistances^32^. In particular, our model treats the thermal contact resistance between needle and sample as negligible in the analysis, which is a typical treatment for 3-omega measurements of liquids and soft materials^20^ owing to their capacity to reflow and conform to the rigid sensor. Therefore in this work we focus on the sensor’s utility for measuring thermal conductivity, as discussed next.

**Fig. 3:**
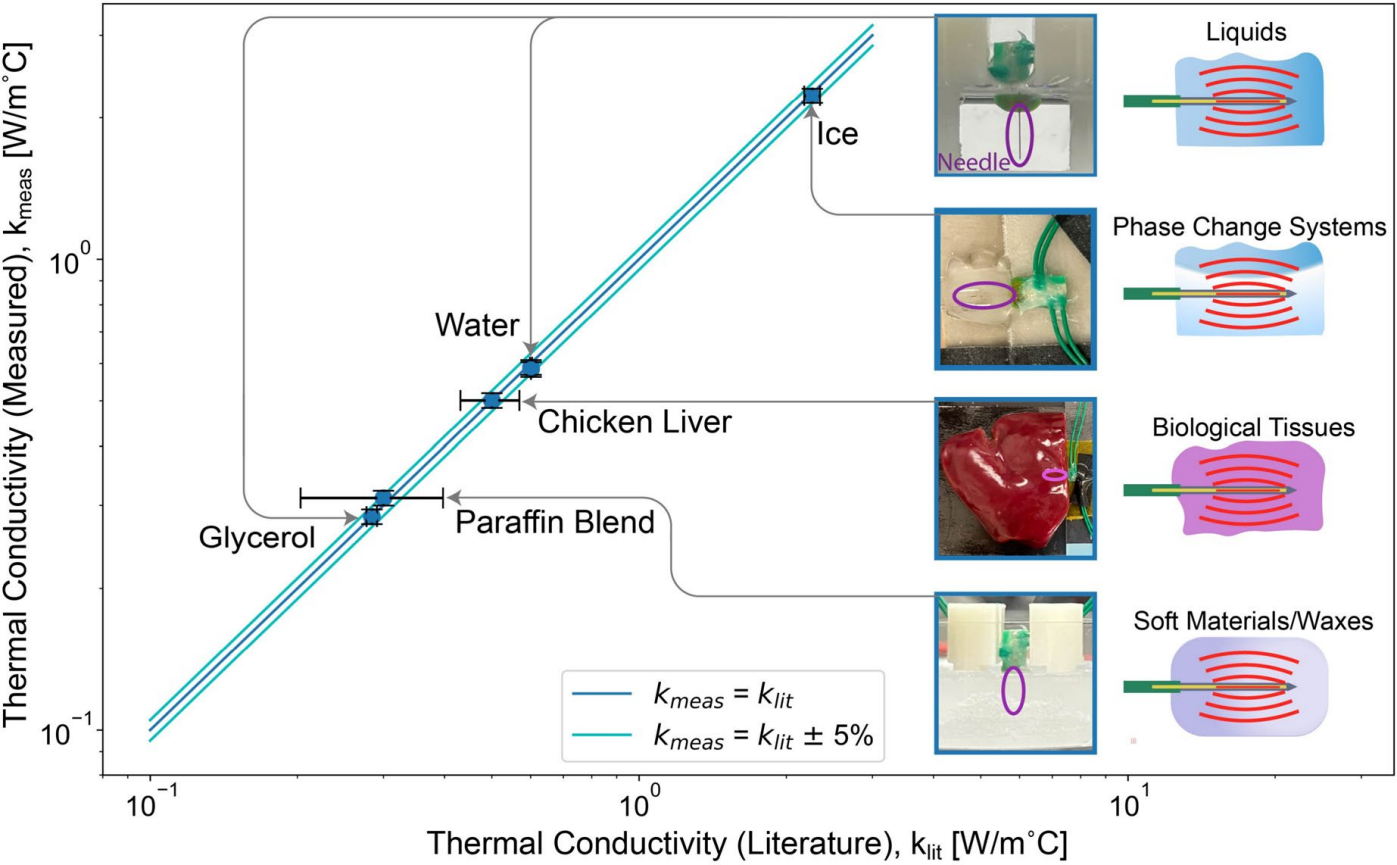
Comparison of measured sample thermal conductivity to literature values for five samples. The lines indicate perfect agreement with literature (dark blue) and agreement to within ±5% (light blue). All measurements are at room temperature (20 - 23 $^{\circ }\hbox {C}$) except for ice at $-12 ^{\circ }\hbox {C}$. Data for liquid water was taken on two different samples, plotted independently and varying by less than 1%. Literature data are sourced from Ramires et al./NIST for liquid water^28^, Harvey/NIST for ice^33^, Bioucas et al. for glycerol^31^, Bianchi et al. for tissues^34^, and Kenisarin et al. and Sobolciak et al. for paraffin wax blends^35,36^. For liver and paraffin blends, the point indicates the median value from literature, and horizontal error bars represent one standard deviation thereof. The vertical error bars of measured data represent the propagated uncertainty of the error sources identified in Supplemental Table 3.

### Sensor performance evaluation

Figure 3 shows the measured thermal conductivities of the chosen samples as compared to literature values. Each data point represents the average of 5 measurements, with deviations between measurements typically differing by less than 1%. All measurements fall within ± 3% of their respective reference values from the literature.

We consider three of the samples – deionized liquid water, ice, and glycerol – to be reference materials, for which highly reliable values found in literature allow for calibration and evaluation of the method. As mentioned above, the value of the parasitic heat loss $R_\text {shank}$ was set to minimize the error for the case of glycerol, therefore guaranteeing extremely close agreement with its literature value (within 0.001 $\hbox {W/m}^{\circ }\hbox {C}$ of the accepted value of 0.284 $\hbox {W/m}^{\circ }\hbox {C}$^31^). After this step there are no more adjustable parameters and we achieve excellent agreement with accepted values for the cases of water and ice as well. To demonstrate the repeatability of the measurement liquid water was sampled twice, with different sensors. The resulting values of 0.589 and 0.584 $\hbox {W/m}^{\circ }\hbox {C}$ are in mutual agreement of better than 1%, and are each within 3% of the reference value of 0.601 $\hbox {W/m}^{\circ }\hbox {C}$) for $T=21^{\circ }\hbox {C}$^28^. Similarly, for ice, the measured value of 2.23 is within 2% of the expected value of 2.26 $\hbox {W/m}^{\circ }\hbox {C}$. This can be considered a very good level of agreement between measurement and literature values, including in the context of round-robin measurements of a standard sample of rigid semiconductor^37^ and comparable to the error of handbook values for liquid thermal conductivities^38^.

An exploration on the contribution of individual sources of uncertainty on this measurement and a table of residuals from the model fits are given in Supplementary Information (see: Supplemental Tables 2 and 3) and are combined to create the propagated uncertainty used in the error bars in Fig. 3. From these potential sources of error, we estimate the typical propagated uncertainty of this measurement to be between 3% and 4%.

The other two samples – chicken liver and paraffin wax – have wider ranges for the literature thermal conductivity. The thermal conductivity of *ex vivo* tissues varies substantially in the literature. Measurements of chicken liver are not available, but room temperature measurements of human, porcine, bovine, and ovine livers typically range from 0.48--0.53^34^ $\hbox {W/m}^{\circ }\hbox {C}$ (represented in Fig. 3), with outliers as low as 0.35 $\hbox {W/m}^{\circ }\hbox {C}$^39^ and as high as 0.58 $\hbox {W/m}^{\circ }\hbox {C}$^40^. At 0.506 $\hbox {W/m}^{\circ }\hbox {C}$, our measurement is within 2% of the median value of this data.

In this study we use a blended paraffin wax for biological applications; according to the vendor SDS, it is composed of 80–85 wt% paraffin, 15–18% butylated hyrdoxytoluene, and 3–5% polyisobutene. The thermal conductivity of this compound has not to our knowledge previously been measured, but our *k* result aligns well with previous studies of other composites of paraffin wax ($\ge 50\%$ w/w) and organic molecules^35,36^ (indicated as the literature value range in Fig. 3). Additionally, the thermal conductivity of paraffin wax is variable in general^35^, and our result also sits within typical values found for pure paraffin wax (for which typical measurements find thermal conductivities of 0.2 to 0.3 $\hbox {W/m}^{\circ }\hbox {C}$).

## Discussion

Here we present the design, modeling, verification, and application of a needle-form 3-omega sensor for measuring the thermal conductivity of soft materials, including those undergoing phase transformations and biological tissues. The sensor and associated analysis is highly repeatable and yields *k* values in excellent agreement (3% or better) with literature reference values. As such, we believe that this sensor could be readily integrated for *in situ* measurements of diverse soft materials and biological tissues, including those undergoing phase transformations.

The present sensor implementation samples a region that is roughly 3--4 mm long and 0.1–0.5 mm in radius in the interior of a soft solid or liquid sample, as determined by the size of the “*T* sensor” region in Fig. 1b plus the surrounding sample within approximately one penetration depth $\delta _p$. Thus the probed volume depends on the sensor size, measurement frequency, and the sample’s thermal conductivity and heat capacity (recall Eq. 1). As such, measured samples should exceed this total volume. For smaller samples, the extent of the radial sampling can be controlled by simple experimental conditions (viz. the frequency of the input current).

We also consider various effects that can limit the applicability of this sensor. The external surface of the sensor is comprised entirely of silicon and silicon-based dielectrics, which allows for a wide range of sample compatibility. This method assumes internal placement of the sensor, which requires a soft or liquid sample state when the sensor is inserted. The analysis model neglects thermal contact resistance, so the needle should be in good thermal contact with the sample at the time of measurement. While we expect this to be well satisfied for liquids and soft compliant samples due to their conformability, and we also found good results for ice after freezing, this thermal contact requirement should be questioned if measuring the solid phase of materials that shrink upon freezing, and should also be considered in phase change materials that undergo large volume changes which were outside the scope of the present study. For soft solid samples, the potential for sample damage during needle insertion, and the resulting possible change of local properties, can also be considered. For gently inserted needles as in the present study we expect the zone of such possible damage to be limited to a volume not much bigger than the needle itself. For particularly thin samples (or for surface measurements), the needle 3-omega sensor could be pressed on the exterior of the sample and then covered with a thermally insulating backing layer, as seen in other 3-omega measurements of soft materials^23,24^.

Placing the thermal probe within the sample’s interior (Fig. 1b) rather than on its surface as in the traditional 3-omega method (Fig. 1a)^13–15^ offers some fundamental advantages. First, nearly all of the needle-form sensor’s joule heating flows usefully into the sample to be probed, while in the traditional supported 3-omega configuration a substantial fraction (can be $\sim$50% or more) of the sensor’s heat is lost into the supporting substrate, which reduces the measurement sensitivity and requires additional calibration. Furthermore, the needle scheme can be more robust against confounding effects of parasitic radiative and convective heat transfer from the sample surface, since the probe itself measures the interior properties away from the surface; this can be particularly important for measurements of samples far from ambient temperatures, such as for high-temperature thermal storage media^2^ and cryobiology^1^. Finally, placing the probe well within the sample interior also helps ensure the freshness of the material measured, reducing the potential confounding effects of surface oxidation, aging, or contamination, which can be particularly important for biological tissues and other air-sensitive materials.

We further note that the fabrication process for these sensors is already well developed for a class of neural probes. Therefore, these sensors would also be suitable for thermal characterization of *in vivo* tissues, a biothermal measurement configuration for which little data is readily available and which has been shown to behave differently from *ex vivo* samples^41,42^.

The sensor provides a novel and distributable method for measuring thermal conductivity in an increasingly critical class of materials; it is able to sample small volumes of soft materials and biological tissues under experimental or operational conditions. In demonstrating such a sensor, we hope to further efforts to measure a wide array of soft functional materials and to advance the ability to model and optimize their thermal behavior.

## Methods

### Preparation of needle-form 3-omega sensor

Sensors were procured from NeuroNexus, Inc. based on our own custom design. Specifications were transmitted to NeuroNexus, who fabricated the sensors based on an adaptation of their small animal electrode arrays.

Once received, the sensors were prepared by attaching four AWG24 insulated copper wires to the contact pads on the printed circuit board of the as-received sensor using MG Chemicals 8331D Silver Conductive Epoxy. After allowing the epoxy to set, Loctite Super Glue was applied for additional mechanical stability and allowed to set. Finally, Sil-Poxy silicone adhesive was applied to both sides of the connecting printed circuit board to improve the water resistance of the electrical contacts.

Before the soft-sample measurements of Figure 3, sensors were calibrated by measuring their electrical resistance vs. temperature. This was done by measuring the voltage drop across the sensor with a very small input current (0.99 mA, much smaller than the 8.07 mA used in the main 3$\omega$ experiments) in a well-controlled thermal environment over a temperature range spanning $\sim 30^{\circ }\hbox {C}$. See SI for details.

Sensors were held by a 3-D printed scaffold for liquid and phase change samples (water, glycerol, paraffin), and held by electrical tape on solid substrates for ice and chicken liver. See photos at the right of Fig. 3.

### Experimental configuration

See Fig. 1.d. Data were taken using a Stanford Research Systems SR830 lock-in amplifier, which also served as a sinusoidal voltage source. The lock-in’s sinusoidal voltage was fed into a custom circuit which converts the voltage into a current source; this circuit also facilitates background subtraction to cancel out nearly all the 1$\omega$ signal by impedance matching with an IET PRS-330 Programmable Resistance Substituter. The circuit’s current output drives the $(I+, I-)$ pads depicted in Fig. 1.b and thereby provides uniform joule heating along the entire shank of the needle, while the voltage leads $(V+, V-)$ tap only the furthestmost 2 mm portion of the needle, labeled “Temperature Sensor” in the Figure.

Experimental controls were run from a Python script, which performed the 1$\omega$ signal cancellation before sweeping frequency logarithmically for 14 in-phase and out-of-phase data points from 118 Hz to 2.001 Hz. The system was given 45 seconds to equilibrate at each frequency step, and measurements are 30 second numerical averages taken after equilibration. The heating current was set using the lock-in amplifier’s voltage sine-out value and the custom *V*-to-*I*, circuit and measured independently on an Agilent 34401 A Multimeter. The current for all 3-omega measurements was 8.07 mA.

Room temperature samples were measured between $20^{\circ }\hbox {C}$ and $23^{\circ }\hbox {C}$. To ensure thermal equilibrium samples other than ice were placed in a bath of liquid water at room temperature and allowed to equilibrate for 2 hours. Ice was measured at $-12^{\circ }\hbox {C}$. Temperature measurements were taken with a fine gauge Omega T-type thermocouple inserted into the volume of the measured sample.

### Sample preparation

Glycerol (Sigma 5516) and deionized water (Thermo Fisher 751-628) filled a small petri dish into which the sensor was inserted. Liquid samples were approximately 20 mL in volume. Paraffin wax blend (Fisherbrand Histoplast PE 22900700) was heated to $80^{\circ }\hbox {C}$ and the sensor was inserted in the liquid state. After this, the sample was allowed to cool to room temperature overnight before measurements were taken in the solid phase. Ice samples were taken by suspending the sensor above a cold plate, onto which deionized water was dropped to form a sample mass of appropriate size that encompassed the needle-form sensor. Chicken livers were sourced from a local grocer. The sensor was gently inserted into the center of the liver from the side of the tissue and placed on a glass slide.

### Numerical model

Data were analyzed through a non-linear optimization algorithm (fmincon in matlab) which called the 3-D simulated version of the system modeled in comsol. Details of the comsol model can be found in Supplemental Figures 2 and 3 and Supplemental Table 1 of the Supplementary Information.

## Supplementary Information

Supplementary Information.

## Data Availability

Data will be made available upon reasonable request to the corresponding authors.
