# Supplementary material for: A needle-form 3-omega sensor for thermal conductivity measurements of soft materials and biological tissues
Source: Sci Rep. 2025 Nov 28;15:42670. doi: 10.1038/s41598-025-26808-1 (PMC12663592; doi:10.1038/s41598-025-26808-1)
Supplement: Supplementary file 1 — Supplementary Information. [file 41598_2025_26808_MOESM1_ESM.pdf]

Supplementary Information for "A needle-form 3-omega sensor for thermal characterization of soft materials and biological tissues"

Spencer P. Alliston[1,*] **and Chris Dames**[1,*]

[1]University of California, Berkeley, Department of Mechanical Engineering, Berkeley, CA, 94720, USA
[*]spencer.alliston@berkeley.edu (Spencer P. Alliston), cdames@berkeley.edu (Chris Dames)

Included in this document

1. Supplemental Figure 1: Temperature Coefficient of Resistance Measurements
2. Supplemental Table 1: COMSOL Model Inputs
3. Supplemental Figure 2: Schematic of Model Inputs
4. Supplemental Figure 3: Sensitivity Study
5. Supplemental Figure 4: Individual 3-omega Measurements for All Samples
6. Supplemental Table 2: Residuals of 3-omega Model Fits
7. Propagated Uncertainty for 3ω Measurements
8. Supplemental Table 3: Error Contributions to Thermal Conductivity Measurements
9. Penetration Depth and Convective Heat Transfer
10. Supplemental Table 4: Rayleigh Number Calculation

Temperature Coefficient of Resistance Measurements

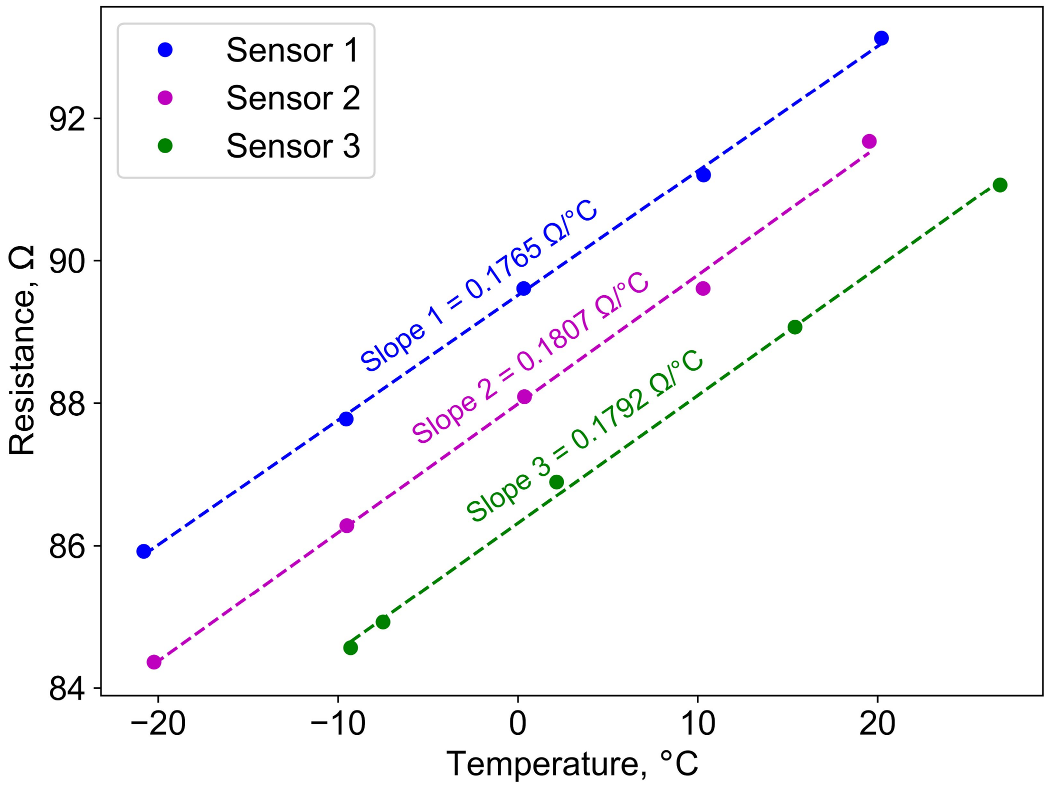

Supplemental Figure 1. Fitting lines for the $\frac{dR}{dT}$ component of the temperature coefficient of resistance for each of the sensors used in this study. Dashed lines indicate simple linear regressions of the data, with the slope of the fit indicated in units $\Omega/°\text{C}$.

The temperature coefficient of resistance term in Eqn. 2 of the main text is calculated by $\alpha = \frac{1}{R_0}\frac{dR}{dT}$. R_0 is the electrical resistance of the sensor line at room temperature and is measured during experimentation in order to cancel the 1ω signal. $\frac{dR}{dT}$ must be calibrated separately for each sensor. Temperature control was implemented with a Fluke 6330D calibration bath. Data from the characterization can be seen in Fig. 1. Fits are simple linear fits of the resistance data, for which the slope is $\frac{dR}{dT}$. From this, α can be determined.

COMSOL Model Inputs

Input	Value	Details
$Q_{straight}$	$0.94{*}I^2R$ [W]	Heat input along shank, using measured I and R. Informed by electrical model.
Q_{bend}	$0.06{*}I^2R$ [W]	Heat input along bend at needle tip, using measured I and R. Informed by electrical model.
k_{sample}	Variable [W/m°C]	Thermal conductivity of sample, used to fit experimental data.
$\rho c_{p,sample}$	Variable [kJ/m^3]	Volumetric heat capacity of sample, used to fit experimental data.
R_{shank}	3,200 [°C/W]	Effective thermal resistance capturing parasitic heat loss along the needle shank. See note below.

Supplemental Table 1. COMSOL model inputs for data analysis model.

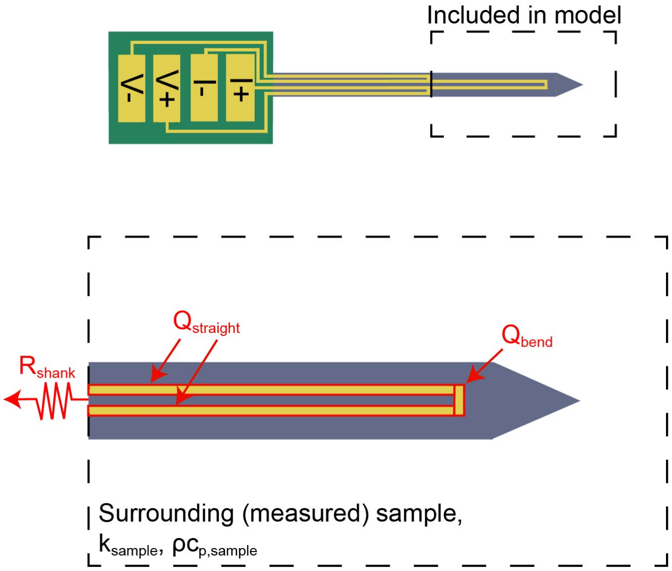

Supplemental Figure 2. Representation of COMSOL simulation extents and inputs, shown in Supplemental Table 1, as demonstrated on needle schematic from Figure 1.b.

The model is a 3D numerical COMSOL model which covers the length of the temperature sensing portion of the sensor (i.e. the last 2 mm of the length) plus ∼0.75 mm of surrounding sample in all directions. The needle geometry in the model is based on fabrication schematics from NeuroNexus, Inc. The gold line is modeled with a thickness of 240 nm and a line width of 20 µm. The thermal properties of the line are assumed to be those of pure gold. The surrounding needle is comprised of a silicon substrate and a dielectric layer. The silicon substrate is 15 µm thick and 100 µm wide. The dielectric layer is 10 µm thick. The pointed tip of the needle extends 100 µm beyond the metal line. The dielectric layer itself is composed of silicon dioxide, silicon nitride, and silicon carbide according the vendor. However, as the relevant thicknesses and compositions of the dielectric layer were not shared, we took the dielectric layer and the silicon substrate to both have the thermal properties $(k, \rho C_p)$ of pure crystalline silicon. We note in Supplemental Table 3 that the measurement is highly insensitive to the thermal conductivity of the needle.

The model is run using a frequency-domain solution with periodic heat inputs. For numerical efficiency, the needle was modeled as a half-needle with mirror symmetry (adiabatic boundary condition) about the plane containing the needle center line and extending out of the page in Supplemental Figure 2. The thermal contact resistances were assumed to be negligible between the gold line and the substrate/dielectric as well as between the needle and the sample. Of the remaining five boundaries of the simulation box, four are set to be constant temperature at the measured experimental temperature T_{env}, corresponding to the far-field thermal conditions of the sample; this is appropriate since the simulation domain extends significantly farther in those directions (∼0.75 mm) than the scale of the thermal penetration depth at the frequencies of interest.

The final boundary condition is for the left-hand face of the simulation domain indicated in Supplemental Figure 2. Here, as noted in the main text, we must account for the additional parasitic heat loss through the shank of the needle itself, indicated conceptually as R_{shank} in Supplemental Figure 2. This was accounted for through the addition of a linear thermal resistor out of the model domain, uniformly contacting the cross-section of the needle there (25 µm thick by 50 µm half-width), and with its other end anchored to the same environmental T_{env}. The rest of that left-hand face of the simulation domain was modeled

as adiabatic. The value R_{shank}=3200 °C/W was determined by matching the ΔT_{AC} vs. ω frequency sweep for the measured sample of glycerol with its thermal conductivity set to the literature value. As a consistency check, this empirically fit R_{shank} is of the same order of magnitude as $\frac{l_{\text{shank}}}{k_{\text{shank}} A_{\text{c,shank}}}$. This value was kept constant in this study based on the measurement of glycerol, and performs well across the range of sample thermal conductivities reported in the main text. Because the relative impact of the parastic heat losses through R_{shank} should become less as the sample thermal conductivity gets higher, we believe that this R_{shank} value would continue to provide reliable results for samples of higher k , including above the largest values of k reported in the main text for ice. Conversely, the k measurement becomes more sensitive to R_{shank} for lower sample thermal conductivities. More characterization would likely be needed to reliably implement such a sensor for samples that are significantly lower in thermal conductivity, such as aerogels.

Model inputs are shown in Supplemental Table 1. Supplemental Figure 2 helps explain how certain model inputs are determined from the overall needle geometry given in main text Figure 1.b I is the measured current from the current source, and R is the measured 4-point probe resistance of the sensing portion of the line (which is the same section of the line in the COMSOL model). The corresponding I^2R joule heating is implemented as volumetric heat sources along the heater line. The relative weights of the "straight" portions (47% each for the upper and lower runs depicted in Supplemental Figure 2) and the "bend" portion (6%) are informed by the needle geometry from the vendor. The thermal conductivity and volumetric heat capacity are fit simultaneously by a two-parameter fitting algorithm implemented in MATLAB.

The resulting COMSOL model solves for 211,460 degrees of freedom and typically runs for $\sim$67 seconds on a lab desktop computer to calculate the simulated equivalent of a full frequency sweep worth of experimental data.

Sensitivity Study

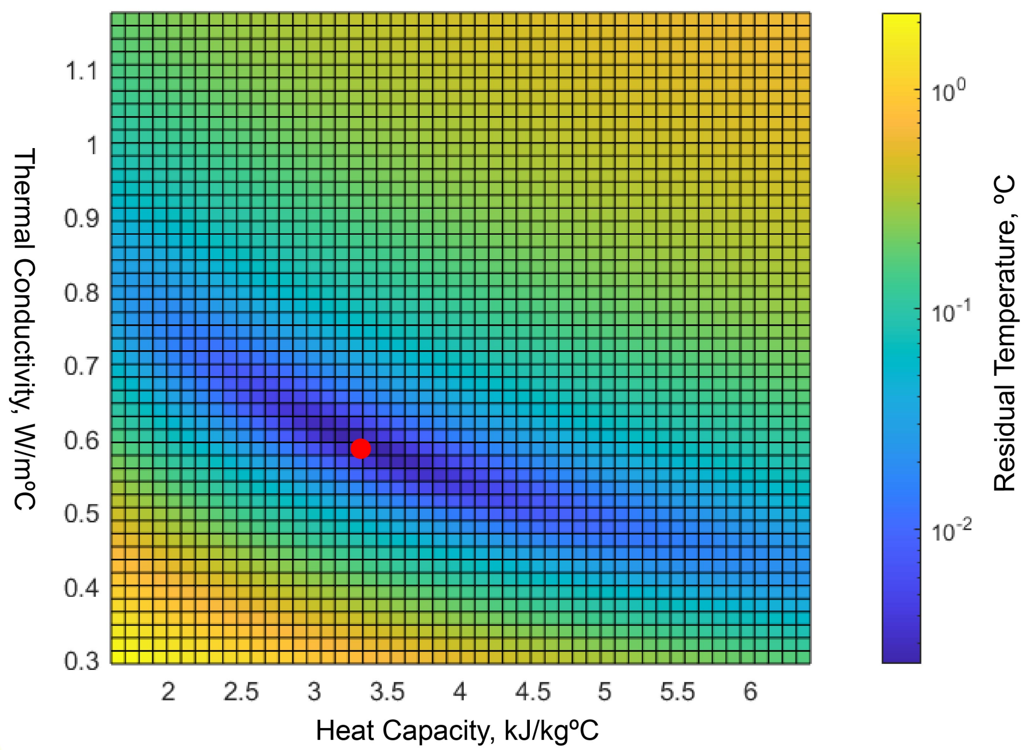

Supplemental Figure 3. Sensitivity study showing the effects of thermal conductivity and heat capacity on the residual (colormap) between model and experiment for a ΔT_{AC} vs. ω frequency sweep of a representative sample of deionized water. The absolute minimum is indicated by the red dot, at coordinates (k_{fit}=0.588 W/m°C , c_{fit}= 3.203 kJ/kg°C). It can be seen that in the neighborhood of the minimum, the model is significantly more sensitive to thermal conductivity than it is to heat capacity.

The sensitivity study in Supplemental Figure 3 shows the reduced sensitivity to heat capacity for this sensor as applied in this study. This, in addition to possible sources of error mentioned in the main text, led us to discount the heat capacity values measured in this study. We hypothesize that, with careful experimental considerations and characterization of the sensor and its thermal interactions with the surroundings (including the contact resistance between the sensor and the sample), this measurement could measure both parameters simultaneously.

We do find that the measurement performs better if the heat capacity is allowed to vary (even though the resulting heat capacity values are significantly erroneous). If the heat capacity is constrained (e.g. to the literature value), the thermal conductivity measurement becomes less accurate. However, we do not find that the deviation of measured heat capacity from expectation has any correlation with the accuracy of the thermal conductivity measurement.

Individual 3-omega Measurements for all Samples

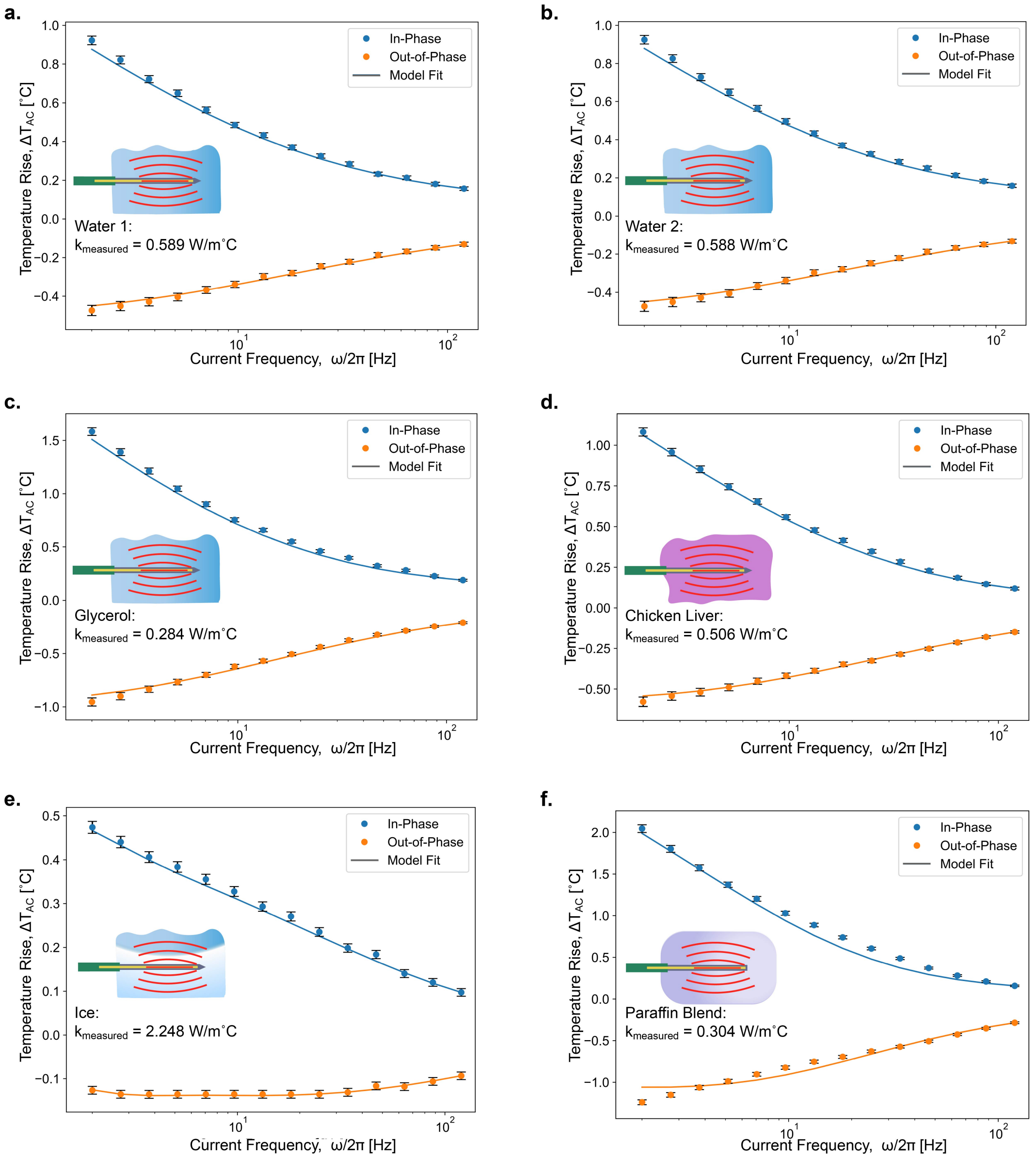

Supplemental Figure 4. Fits and thermal conductivity values for individual 3-omega measurements of a) water, sample 1, b) water, sample 2 (repeated from Fig. 2b of the main text), c) glycerol, d) chicken liver, e) ice, and f) paraffin blend. The inset drawings depict the needle in the sample, with red lines representing the thermal waves from periodic heating by 8.07 mA electrical current.

Sample	RMS Residual Temperature	RMS Fractional Residual
Water 1	0.017 [°C]	0.037
Water 2	0.017 [°C]	0.039
Glycerol	0.036 [°C]	0.055
Chicken Liver	0.015 [°C]	0.037
Ice	0.008 [°C]	0.031
Paraffin Blend	0.075 [°C]	0.107

Supplemental Table 2. RMS Residual Temperatures and RMS Fractional Residuals of fits depicted in Supp. Fig. 4. Residuals are given to better evaluate the fits given, and fractional residuals allow for better comparison between samples. It can be seen that, while most of the fits are quite similar to one another in fractional residuals and have temperature residuals less than 0.04°C, the fit for the paraffin blend is not as good as the others, with a temperature residual exceeding 0.07°C.

Propagated Uncertainty for 3ω measurements

In Figure 2.b, the error bars are the propagated uncertainty of the measurement, calculated by:

$$\sigma_{\Delta T_{AC}} = \Delta T_{AC} * \sqrt{\left(\frac{\sigma_{V_{3\omega}}}{V_{3\omega}}\right)^2 + \left(\frac{\sigma_I}{I}\right)^2 + \left(\frac{\sigma_{dR/dT}}{dR/dT}\right)^2} \quad (1)$$

where $\sigma_{\Delta T_{AC}}$ is the propagated uncertainty as plotted in Figure 2.b, ΔT_{AC} is the measured temperature oscillation, $\sigma_{V_{3\omega}}$ is the noise of the lock-in amplifier (6 nV/$\sqrt{\text{Hz}}$ per SRS), $V_{3\omega}$ is the measured voltage, σ_I/I is the relative error of the V-to-I converting circuit taken as 1%, $\sigma_{dR/dT}$ = 0.005 Ω/°C is the standard error of the fit shown in Supplemental Figure 1, and dR/dT is the relevant slope of the fits shown in Supplemental Figure 1. For our conditions the $\left(\frac{\sigma_{dR/dT}}{dR/dT}\right)$ term has the greatest contribution to the overall uncertainty in $\sigma_{\Delta T_{AC}}$.

Impact of Individual Uncertainties on Thermal Conductivity Measurements

In addition to the standard uncertainty of the thermal conductivity measurements, as calculated from the variability of 5 repeated measurements and depicted by the error bars of Fig. 3, individual sources of error were also evaluated for their impact on the final measurement.

Here, we manipulate several variables, each in isolation, by the amounts given in Supplemental Table 3 and report the change in predicted thermal conductivity averaged across all samples. The examined parameters included those due to errors in the sensor geometry, the thermal behavior of the needle, and the contribution of the $\frac{dR}{dT}$ component of the temperature coefficient of resistance, which was the dominant source of error in the propagated uncertainty $\sigma_{\Delta T_{AC}}$.

It can be seen from the table that the propagated uncertainty is of comparable magnitude as the difference between expected thermal conductivity values and measured thermal conductivity values. This supports the validity of the reported 3% deviation from expectation; the errors from Supp. Table 3 combine in quadrature to 3.6%, which is used as the representative error for all points in Fig. 3. We further note that these values represent relatively high parameter errors (using high errors for fabrication and the highest standard error of the TCR fits, which was 20-40% higher than for the other sensors) and that the performance of the implemented measurement was consistent across 3 sensors and 6 samples.

Parameter	Parameter Error	Error in Fit Sample Thermal Conductivity	Notes
Length of heater line	1%	0.6%	Estimated microfabrication error
$\frac{dR}{dT}$ (see: Supp. Fig. 1)	2.9%	2.8%	Highest Std. Err. from fits
Needle Thermal Conductivity	30%	0.6%	k_{needle} within simulation domain
R_{shank} (see: Supp. Fig. 2)	10%	2.1%	Sensitivity to fit heat loss parameter

Supplemental Table 3. Examination of individual sources of error and their potential impact on measured thermal conductivity values.

For example, we considered the effect of perturbing up and down by 10% from its nominal value of 3200 °C/W. For each of the six datasets depicted in Supp. Fig. 4, we generate two more COMSOL models, one using R_{shank}=0.9*3200 °C/W, and the other using R_{shank}=1.1*3200 °C/W. These are then fit for thermal conductivity, and the difference between this new thermal conductivity (e.g., 0.293 W/m°C for water with R_{shank}=0.9*3200 °C/W) and the original (e.g. 0.284 W/m°C for water with R_{shank}=3200 °C/W) and the original is averaged across all samples for the 2.1% k error in response to a +-10% change in R_{shank}.

Similar perturbations and COMSOL re-analysis were performed for each of the other rows of the table.

Thermal Penetration Depth and Convective Heat Transfer Considerations

The thermal penetration depth is determined by the heating frequency and the thermal diffusivity of the sample, as defined by Eq. (1) of the main text. As such, the frequency range used for a given experiment should be chosen to ensure that the sample volume probed by the heat penetration is appropriate for that particular sample.

In this study, the lower bound of frequency was generally limited by one of two factors. For our smaller samples, which tended to be the solids, the lowest frequencies were chosen to ensure that δ_p still remains well inside the sample, so that the sample can be approximated as semi-infinite. To measure thinner or more conductive samples, higher values of the lower-bound frequency can be selected to avoid the thermal waves feeling the boundary of the sample.

Liquid samples, even very large ones, have an additional constraint due to the possibility of natural convection, and so the lower frequency should also be high enough to ensure that convection remains suppressed, as discussed further below.

For all samples the upper bound of the frequency sweep should be set such that the needle temperature rise still depends significantly on the thermal conductivity of the sample (as opposed to becoming dominated only on the thermal properties of the needle). As a guideline we expect this to be reasonable as long as δ_p is two or three times larger than the thinnest needle cross-section dimension which is 25 µm.

It is well known that natural convection is well suppressed for conditions of sufficiently low Rayleigh number:

$$Ra_L = \frac{\rho \beta \Delta T L^3 g}{\eta \alpha_T} < 1708,$$

where Ra_L is the Rayleigh number, ρ is the density of the fluid, β is the fluid's volumetric thermal expansion coefficient, ΔT is the temperature drop between the needle and the bulk fluid, L is the characteristic length scale over which ΔT occurs which here we take as $L \approx \delta_p$ (from Eqn. 1), g is the acceleration of gravity, η is the dynamic viscosity of the fluid, and α_T is the thermal diffusivity of the fluid.[1]

This critical Ra_L condition can be evaluated for two representative liquids at the lowest oscillation frequencies (which is the worst-case condition because it maximizes both ΔT and L). Using the values given in Table 4, we find $Ra_{\mathrm{L,water}} = 179$ and $Ra_{\mathrm{L,glycerol}} = 0.84$. Since these are both far below the critical value of 1708 we conclude that natural convection can be very safely ignored in these conditions.

The fluids and conditions in this study are safely far below the critical Rayleigh number, and we would expect similar for most liquids at room temperature. However, for fluids that have particularly high thermal conductivity and/or particularly low viscosity (e.g. gases), this should be reevaluated. By lowering the total heat input, and therefore ΔT, and increasing the frequency, and therefore lowering L, the Rayleigh numbers could be further lowered for these cases if needed.

Parameter	Water	Glycerol
ρ [kg/m^3]	998	1271
β [1/°C]	2.14×10^{-4}	6.01×10^{-4}
ΔT [°C]	0.93	1.58
L [m]	2.37×10^{-3}	1.91×10^{-3}
g [m/s^2]	9.81	9.81
η [kgm/s]	1.01×10^{-3}	0.98
α_T [m^2/s]	1.43×10^{-7}	1×10^{-7}
Ra_L [1]	179	0.84

Supplemental Table 4. Rayleigh number calculation for water and glycerol. For sufficiently low Rayleigh numbers, convective heat transfer can be neglected. Where needed, materials data for water was sourced from NIST[2]. Data for glycerol was sourced from Blazhnov et al.[3] for density and thermal expansion, Ferreira et al.[4] for viscosity, Bioucas et al.[5] for thermal conductivity, and NIST[2] for the heat capacity.

References

1. Bergman, T. L., Lavine, A. S., Incropera, F. P. & DeWitt, D. P. *Introduction to heat transfer* (John Wiley & Sons, 2011).
2. Linstrom, P. & Mallard, W. (eds.) *NIST Chemistry Webbook, NIST Standard Reference Database Number 69* (National Institute of Standards and Technology, Gaithersburg MD, 20899, retrieved October 1, 2025).
3. Blazhnov, I. V., Malomuzh, N. P. & Lishchuk, S. V. Temperature dependence of density, thermal expansion coefficient and shear viscosity of supercooled glycerol as a reflection of its structure. *The J. chemical physics* **121**, 6435–6441 (2004).
4. Ferreira, A. G. *et al.* The viscosity of glycerol. *The J. Chem. Thermodyn.* **113**, 162–182 (2017).
5. Bioucas, F. E., Koller, T. M. & Fröba, A. P. Thermal Conductivity of Glycerol at Atmospheric Pressure Between 268 K and 363 K by Using a Steady-State Parallel-Plate Instrument. *Int. J. Thermophys.* **45**, 1–12, DOI: 10.1007/s10765-024-03347-x (2024).
